# Social Determinants of Seeking and Reaching Injury Care in South Africa: A Community-Based Qualitative Study

**DOI:** 10.5334/aogh.4003

**Published:** 2023-01-27

**Authors:** Eyitayo O. Owolabi, Karen Ferreira, Samukelisiwe Nyamathe, Agnieszka Ignatowicz, Maria Lisa Odland, Abdul-Malik Abdul-Latif, Jean C. Byiringiro, Justine I. Davies, Kathryn M. Chu

**Affiliations:** 1 Centre for Global Surgery, Department of Global Health, Faculty of Medicine and Health Sciences, Stellenbosch University, Cape Town, South Africa; 2 Centre for Health Promotion and Disease Prevention, Edson College of Nursing and Health Innovation, Arizona State University, Phoenix, Arizona, USA; 3 Institute of Applied Health Research, University of Birmingham, Birmingham, United Kingdom; 4 Volta Regional Health Directorate, Ghana Health Service, Ghana; 5 School of Medicine and Pharmacy, Department of Surgery, University of Rwanda, Rwanda; 6 Faculty of Health Sciences, Medical Research Council/Wits University Rural Public Health and Health Transitions Research Unit, University of Witwatersrand, Johannesburg, South Africa

**Keywords:** Injury, Delays to care, Barriers, Social Determinants of Health, Health-seeking behaviour, South Africa

## Abstract

**Background:**

Timely access to quality injury care saves lives and prevents disabilities. The impact of social determinants of health on the high injury prevalence in South Africa is well documented, however, evidence of their role in accessing injury care is lacking. This study explored the social determinants of seeking and reaching injury care in South Africa.

**Methods:**

This was a qualitative study involving rural and urban patients, community members, and healthcare providers in Western Cape, South Africa. Data were obtained through semi-structured interviews and focus group discussions using an interview guide informed by the four-delays framework. Inductive and deductive approaches were used for thematic analysis.

**Results:**

A total of 20 individual interviews and 5 focus group discussions were conducted. There were 28 males (individual interviews: 13; focus groups: 15) and 22 females (individual interviews: 7; focus groups: 15), and their mean age was 41 (standard deviation ±15) years. Barriers to seeking and reaching injury care cut across five social determinants of health domains: healthcare access and quality; neighbourhood and environment; social and community context; education; and economic stability. The most prominent social determinants of seeking and reaching injury care were related to healthcare access and quality, including perceived poor healthcare quality, poor attitude of healthcare workers, long waiting time, and ambulance delays. However, there was a strong interconnection between these and neighbourhood and environmental determinants such as safety concerns, high crime rates, gangsterism, lack of public transportation, and social and community factors (presence/absence of social support and alcohol use). Barriers related to education and economic stability were less prevalent.

**Conclusion:**

We found a substantial role of neighbourhood, social, and community factors in seeking and reaching injury care. Therefore, efforts aimed at improving access to injury care and outcomes must go beyond addressing healthcare factors to include other social determinants and should involve collaborations with multiple sectors, including the community, the police, the transport department, and alcohol regulation agencies.

## Introduction

Traumatic injury is an important public health problem in South Africa (SA), accounting for 12% of all disability-adjusted life years lost in 2019 [1]. Intentional injury in SA is seven times, and road traffic accidents are double the global rate [2]. Risk factors for injuries are multifactorial but include social and environmental factors such as alcohol use, low socio-economic status, social and cultural norms around violence and injury, organised crimes, road structures (road width, number of access roads, median width), and aggressive driving [3,4,5]. Access to timely and quality injury care is crucial in reducing injury-related morbidity and mortality. A previous study in rural South Africa showed that 40% of injury deaths were avoidable, and 38% of these were due to delays in seeking and reaching injury care [6].

Improving timely access to quality health care after an injury is a multifaceted problem [7]. However, many studies of the issues leading to poor access or providing a solution have focused on care provided at the health facility [8] and neglected socio-economic and cultural factors, which have been shown to influence care access [6,9]. Given recognised inequalities in accessing healthcare, there is increasing attention on the impact of social determinants of health on health-seeking behaviour and health outcomes, especially in countries like SA with a high level of inequality [10]. Social determinants of health (SDoH) are factors related to where a person was born, lives, or works that may influence health, functioning, or quality of life [11]. These include factors such as how much an individual earns, the kind of work they do, their level of education, and their neighbourhood [11]. There are five defined SDoH domains: (1) *healthcare access and quality* which can be influenced by medical insurance, health literacy, and socio-economic status; (2) *social and community context*, including the circumstances in which people live, learn and work, community supports and engagement, social cohesion or discrimination; (3) *neighbourhood and environment* including air and water quality, access to transportation, healthy food, violence and other potential health risks; (4) *education* defined by the highest formal education and qualification of an individual as well as literacy level; and (5) *Economic stability* including factors such as employment status, food insecurity, housing instability and poverty [11,12]. Some SDoH domains can directly impact others. For example, education may directly impact health literacy and economic stability.

The influence of social determinants on the incidence of various health conditions, including infant mortality, HIV/AIDs, and non-communicable diseases, has been shown in SA [13]. Similarly, SDoH effects on the prevalence of injuries in SA and elsewhere have been demonstrated [3,4,14,15,16]. The role of SDoH in seeking and reaching care or health-seeking behaviour is also well documented in research. An understanding of the role of SDoH on health-seeking behaviour for injury care is lacking both in SA and other contexts. Given the high prevalence of injuries in South Africa, the recognition that 40% of avoidable mortality occurs before injured patients reach healthcare facilities [6], and the knowledge that SDoH impacts care access for other conditions, it is important to understand how SDoH impact care seeking after injuries.

Since the causes of injury are multifactorial, they usually occur swiftly, and require timely decision-making and healthcare access to avoid disability and death, this means that the role of SDoH in seeking healthcare for injuries may differ from other health conditions that may be less time-sensitive or with specific causes. Understanding the impact of SDoH on accessing care after injuries will help to determine which SDoH need to be addressed per se and, at what point, to successfully implement interventions to improve access. To the best of our knowledge, no previous research has been published on the role of social determinants in seeking and reaching injury care in SA. Therefore, this study aimed at determining the social determinants of seeking and reaching injury care in South Africa to inform potential interventions and the health system response.

## Methods

### Study design and setting

This was a sub-study within the “Equitable access to quality trauma systems in Lower- and Middle-Income Countries: assessing gaps and developing priorities in Ghana, Rwanda and South Africa” study – a multi-country mixed-method project exploring barriers to timely access to quality care after injury. The overarching study, which summarised the shared barriers to equitable access to timely care across three countries has been published [7]. This current study is a qualitative analysis specifically focused on barriers to seeking and reaching injury care in South Africa using an SDoH lens.

The study was conducted in the Western Cape province of South Africa at an urban (Khayelitsha) and a rural site (Worcester). The majority of South Africans seek healthcare through the public health sector. Khayelitsha is the largest informal settlement of the city of Cape Town with a predominantly black population and an estimated population of 442,721 in 2020 [17]. Interpersonal violence and road traffic crashes are the second and fourth leading causes of mortality in Khayelitsha [18]. Residents access the health sector through a series of community health centres and a district hospital, Khayelitsha District Hospital (KDH). Worcester is a rural town located in the Cape Winelands district with an estimated population of 127,597 [19]. Over half of the population in Worcester are of mixed race and 25% are black Africans [19]. Worcester is served by Worcester Regional Hospital (WRH). In addition, WRH receives referrals from seven other district hospitals from the rural town of Ceres, Caledon, Bredasdorp, Hermanus, Montagu, Robertson, and Swellendam, serving an overall catchment of 600,000 people.

### Study participants and sampling

The study participants were over 18 years of age and recruited through purposive sampling. There were three groups of study participants:

Group 1: People residing in the study sites with a history of any type of injury in the preceding six months who received care in the public health sector. Potential participants were identified through two sources; databases of hospital inpatients and outpatients who had been treated for injury. Participants were invited telephonically by a member of the clinical team to take part in the study or approached in person by the study team if seen in outpatient clinics.

Group 2: Community members from the study sites who served on community health forums, the police force, or community-based non-governmental organizations (NGOs). Potential participants were identified through affiliations with local hospitals, clinics, health NGOs and a satellite campus of the University of Stellenbosch.

Group 3: Healthcare providers involved in various aspects of injury care including medical doctors, surgeons, nurses, public health practitioners, occupational therapists, psychologists, social workers, or emergency medical service providers. These participants were recruited from the healthcare facilities at the two study sites using purposive and snowball sampling.

### Data collection

Data were obtained through individual in-depth interviews (IDIs) and focus group discussions (FGDs). The interviews and focus groups followed a semi-structured format using topic guides which were developed to understand experiences and perceived barriers to care after being injured using the four delays framework [9]. The four delays framework described delays to accessing care in four phases: 1) delay in making the decision to seek care, 2) delay in reaching any healthcare facility, 3) delay in receiving definitive injury care, and 4) delay in remaining in care (ongoing care provided as follow-up or rehabilitation to attain optimal function). IDIs and FGDs were done with previously injured patients and FGDs were done with community leaders and healthcare providers.

FGDs were conducted at healthcare facilities. IDIs were either conducted at healthcare facilities or participants’ homes in a private room to ensure confidentiality. Discussions lasted between 45 to 60 minutes (IDIs) and 90 to 120 minutes (FGDs).

They were conducted in one of three South African official languages: English, Afrikaans or Xhosa, by trained investigators (KF and SN) fluent in these languages. IDIs and FGDs were audio-recorded.

### Data analysis

Recordings were transcribed and translated to English. Transcribed data were uploaded into NVivo data management program for coding and analysis [20]. Data was analysed thematically, both inductively and deductively. First, three authors (SN, KF and AI) open-coded a sample of the transcripts to identify explicit examples of perceived barriers to injury care. Additional codes were added based on the Four Delays Framework [21]. These initial codes were discussed among authors and formulated into a coding framework that was used for the analysis of the remaining transcripts. Codes were then categorised into potential subthemes and themes, discussed in a series of analysis meetings, and then refined and labelled.

Further, the identified barriers relating to seeking and reaching injury care were mapped to the five SDoH domains. In addition to describing the findings, the number of barriers mentioned by participants for each of the SDoH domains was counted. Also, the interconnectedness of the SDoH factors for each of the study participants was explored by collecting data on how many other SDoH domains were mentioned by the participants.

### Ethical consideration

Ethical approval for this study was obtained from Stellenbosch University Research Ethics Committee (Approval number: N20/01/010), Western Cape Provincial Department of Health, and the University of Birmingham’s Science, Technology, Engineering and Mathematics Ethical Review Committee (ERN_20-0088). All study participants gave informed consent before data collection, including for audio recordings, and were informed that their involvement in the study would not change their clinical care. All data were anonymised before data analysis.

## Results

A total of 20 IDIs and 5 FGDs were conducted. There were 13 males and 7 females among the IDI participants, equally distributed between the rural and urban settings and with a mean age of 36 (SD±14) years. The gender distribution in the FGDs was equal and the mean age of the FDG participants was 45 (SD±16) years (Table 1).

**Table 1:** Demographic characteristics of the study participants.


	INTERVIEWEES	FOCUS GROUP PARTICIPANTS

Average age (Standard deviation, SD) years	36 (SD±14)	45 (SD±16)

Gender		

Male	13	15

Female	7	15

Place of residence		

Rural	10	13

Urban	10	17

Language		

Afrikaans	10	13

IsiXhosa	10	17


**Figure 1:**
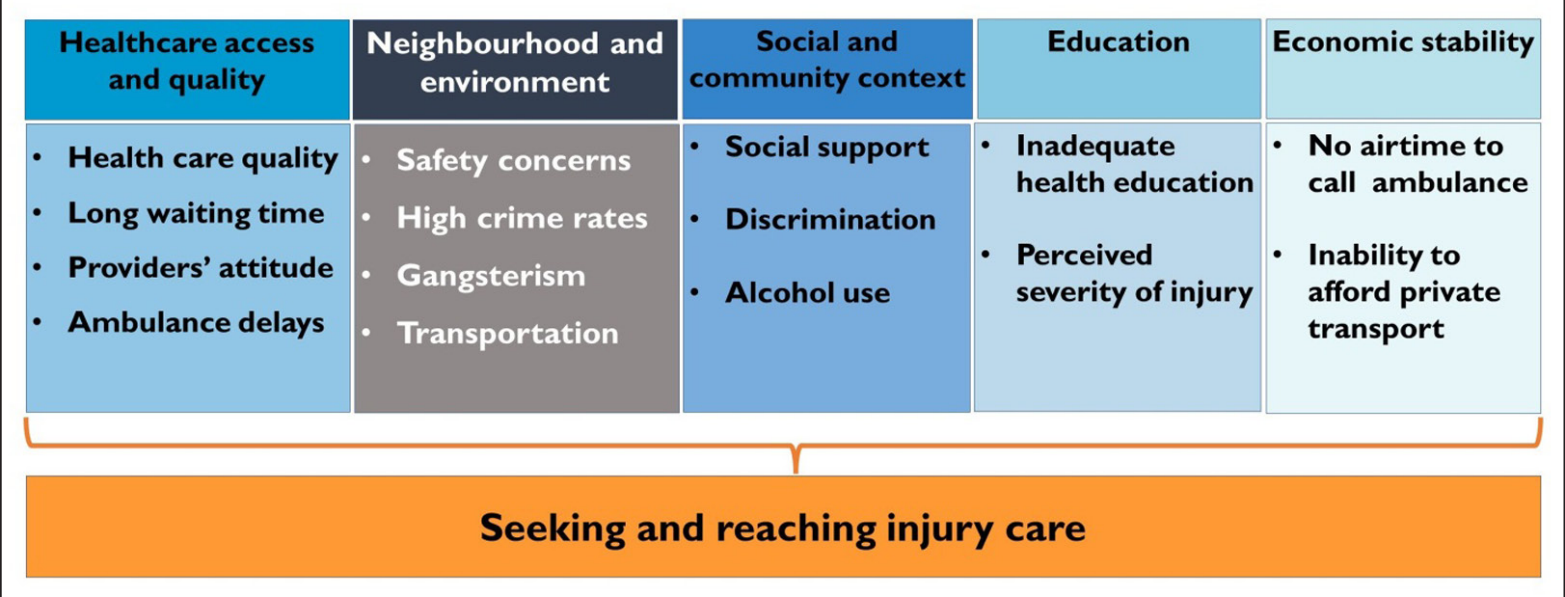
Barriers to seeking and reaching injury care by social determinant domain.

Our study showed several social determinants of seeking and reaching injury care in South Africa. The barriers experienced were related to the five domains of the SDoH framework: healthcare access and quality, neighbourhood and environment, social and community context, education and economic stability (Figure 1).

**Figure 2:**
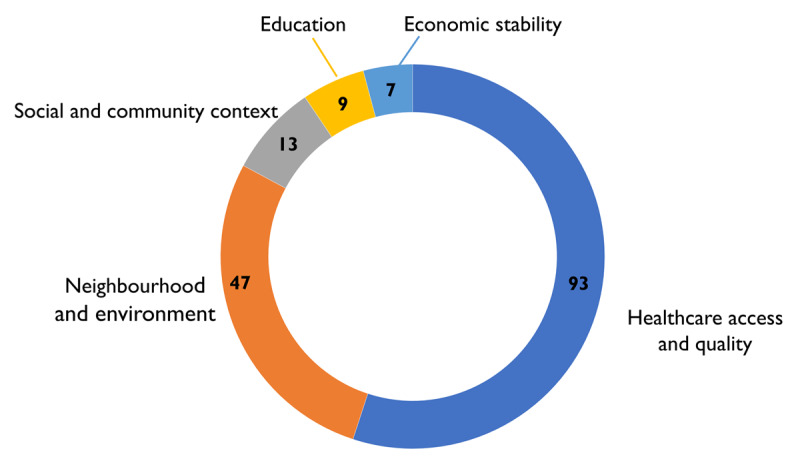
Quantification of barriers to injury care by social determinant domain.

The number of barriers identified by each participant ranged from 1 to 8, with a mean of 4. Barriers related to healthcare access and quality SDoH domain were the most frequently reported (n = 93), followed by neighbourhood and environmental barriers (n = 47). Barriers related to social and community context, education and economic stability were less highlighted (Figure 2).

**Figure 3:**
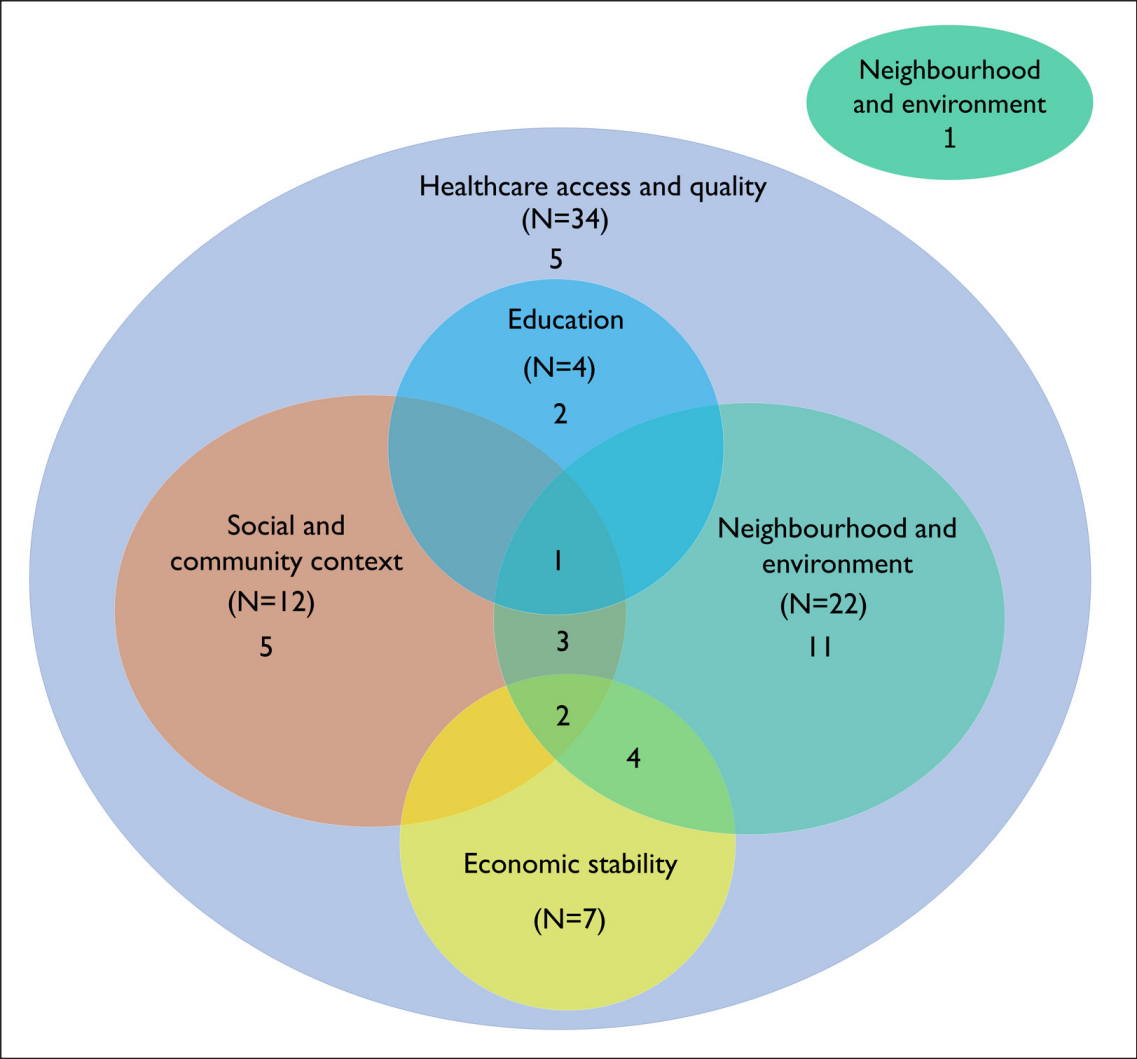
Interconnectedness of social determinants of seeking and reaching injury care.* * Total numbers (N) experiencing barriers are greater than shown for each interaction, as an additional grouping not captured in the Figure including 1 person who experienced overlapping barriers between healthcare access and quality, social and community context, economic stability and education.

As illustrated in Figure 3, barriers to injury care identified by most of the study participants were experienced across many of the SDoH domains. All but one of the participants experienced barriers in the healthcare access and quality domain. Of these, many also experienced neighbourhood and environmental barriers and sometimes in addition to social and community context barriers.

## Healthcare access and quality

The study participants showed that existing health services and perceptions of the healthcare system are important social determinants of seeking injury care.

### Health and Healthcare

Responses in this SDoH domain were mostly around healthcare system quality. Study participants highlighted how previous negative experiences with the healthcare system (either their own or a friend or family member) led to reluctance in seeking care. Some of these negative experiences included the perceived unpleasant attitude of the healthcare workers, perceived poor quality of care rendered (for example, a participant reported just being “poked and prodded” at the health care facilities and was sent home with analgesics) and poor outcomes (death or disability):

*“There are many stories of people who die unnecessarily at the hospital which makes it difficult for anyone to want to go [to the hospital] because of these stories. They [healthcare providers] need to get the community to trust them again.”*

*– 35 years old male, urban*

*“The biggest problem is the staff’s attitude. The nurses get irritated by the very people that they are meant to be helping. It doesn’t matter what time you arrive. From 6 am, they will be angry, and you won’t understand what the reason is.”*

*– Female, unknown age, urban*

### Waiting time

Also, individuals emphasised the perceived or expected long waiting time at the health care facilities and how that may serve as a barrier to seeking injury care. This barrier was noted by participants from both the rural and the urban sites:

*“The issue of people sitting in lines for a very long time is unacceptable. They [the healthcare providers] say it is because they are treating emergencies, but I also felt at the time as if I was an emergency and no one came to explain why I had to wait for that long period of time.”*

*– 29 years old female, urban*

*“I think it depends on whether you have medical aid and can afford to go to a private hospital, but if you have to go to a government hospital and you have to sit and wait from eight o’clock in the morning until four o’clock in the afternoon to be helped. I think anyone in their right mind would prefer not to do that.”*

*– 36 years old male, rural*

## Neighbourhood and environment

Different neighbourhood and environmental factors that could serve as barriers to seeking and reaching injury care were identified and these included the high neighbourhood crimes, gangsterism and transportation issues.

### High crime “red zones”

The features of a neighbourhood where an individual lives play a role in health and health-seeking behaviour. In our study, one predominant neighbourhood factor that influenced seeking or reaching injury care was the high crime rates in the study communities. Some parts of the communities were designated as dangerous or “red zones.” In these areas, participants were reluctant to travel alone or at night, which served as a barrier to seeking care when needed. Also, ambulances are required to have police escorts to enter red zones which delayed reaching care:

*“Travelling a longer distance [to healthcare facility] puts you at risk of being robbed because most of us walk. We do not have cars to drive.”*

*– 35 years old male, urban*

*“Recently, I had to call the ambulance when my baby sustained burns. I waited a long time before they could get here because they usually wait for a police escort.”*

*– 34 years old female, rural*

*“And then if the ambulance does come, in this community, they first need to start at a police station because some places are red territories where ambulances will get robbed and hijacked.”*

*– Male, unknown age, urban*

### Fear of attack by gangs

At times, some injuries may involve gangs and study participants identified how the fear of being attacked in the neighbourhood by gangsters may influence their decision to seek or ability to reach care:

*“It was very dangerous for me to get out of the area, because of gangsterism.”*

*– 19 years old male, rural*

*“And many of the young men that are injured in gangs struggle to get to the hospital. If they walk down there, they go right past the territories of other gangs, so they choose to stay, for their safety.”*

*– Male, unknown age, rural*

### Transportation

The transportation options that were available to injured persons were ambulances, public transport minibuses, taxis or private vehicles owned by family or friends. Due to the high crime rates or the insufficient number of available ambulances, there may be a delay in ambulance arrival, hence several patients would try to access care using public transportation. However, public transport minibuses and taxis were only available at certain hours:

*“Not everyone has access to transport. Most of us struggle. And the ambulance takes some time to come. It takes about an hour and a half, sometimes two hours.”*

*– 36 years old male, rural*

## Social and community context

Social and community context may involve the presence or absence of social support, discrimination within communities or other social vices like alcohol use.

### Social Support

Social and community support are important determinants of health. Good social support and healthy relationships in the community can help overcome some of the uncontrollable social factors such as unsafe neighbourhoods. Participants mentioned different ways in which the community influenced the decision of an injured person to seek care. Community members also assisted at the scene of injury by contacting emergency services and in some cases transported the injured person to the hospital:

*“Initially I was not convinced that I should come to the hospital because I did not think I was seriously injured. But then the people I was with were all saying that I need to come to the hospital because the injuries might bother me later in life.”*

*– 33 years old male, urban*

### Discrimination

Although the participants opined that the community mostly supports each other and that the presence of support may foster seeking care after an injury, it was, however, noted that sometimes offering help or support may be delayed if the injured person is not well known in the community. There was, however, less emphasis on discrimination as a perceived barrier to injury care, as this notion was only shared by a participant who had an accident in a neighbourhood where he was not known.

*“The people in that neighbourhood also do not know me. Immediately after the taxi hit me, it fled the scene. It also depends on if your injury occurred in a neighbourhood where you are well known or if you are a stranger to the community.”*

*– 49 years old male, urban*

### Alcohol use

Unhealthy social behaviour such as excessive alcohol use is another perceived barrier to seeking injury care. Alcohol use and abuse were recognised to impair judgment, and mask the severity of the injury, thus, intoxicated participants only sought help when they became sober and experienced pain related to the injury:

*“I had been drinking so I did not feel any pain. I didn’t pay much mind to it, I got up and then continued my journey home. The next day upon waking up I started feeling my arm in pain. I took [an aspirin] and nothing was helping. The pain just kept getting worse. So, I decided to come in [to the clinic].”*

*– 49 years old male, urban*

## Education

There is evolving evidence of the impact of education on health knowledge, behaviour and outcomes. This in part may be related to health and general literacy which allows more educated individuals to make informed and better health decisions. In this study, we found how an individual’s perception of the injury impacted seeking injury care. Some participants perceived their injury as minor and only sought healthcare when their condition worsened:

*“I snapped my ankle while playing with the kids, and initially I thought it was just a sprain, but the next morning it was still painful and I couldn’t put any weight on it, so I decided to go to the doctor.”*

*– 36 years old male, rural*

## Economic stability

There is a link between poverty, health and health-seeking behaviour. Some participants highlighted the unaffordability of private transport as a barrier to seeking and reaching injury care:

*“Some [private drivers] will decide to charge you R900 knowing that you will not be able to afford the price. Those are the main costs [of seeking care.] It would be transport.”*

*– 22 years old female, rural*

Others indicated the lack of funds to procure airtime to call an ambulance following an injury:

*“Access to a phone is not difficult, but people don’t always have data or airtime [To call an ambulance].”*

*– Male, rural, unknown age*

## Discussion

Timely access to quality injury care is critical to improving outcomes. While previous studies have shown the role of social determinants such as crime, alcohol use, and gang violence on the incidence of injuries [2,3,4], our study described their impact on seeking and reaching care. Some of the identified barriers in this study, such as perceived injury severity and lack of ambulance services, were also highlighted in a study identifying barriers to injury care in Rwanda [9]. A lack of health education and the perceived poor quality of health services received are contributing factors. In addition, we reported neighbourhood crimes, alcohol abuse, and negative perceptions of the health system as barriers to seeking injury care which has not been previously reported, to our knowledge. The ability to address these SDoH, in addition to resolving some of the identified healthcare service concerns, could greatly improve access to care and may increase health-seeking behaviour.

Healthcare factors including poor healthcare quality, healthcare provider’s attitude, long waiting time, and ambulance delays were the most prominent social determinants of seeking and reaching injury care in this study setting. These factors are socially determined by where a person lives and their health insurance status. These factors have negatively impacted community members’ trust in the health system, and the decision to seek injury care. Similar findings have been reported in other settings for other health conditions, including maternity care [22,23,24]. As emphasised by the Institute of Medicine [25], timely and patient-centred care are essential features of quality healthcare. Likewise, patient-centred communication plays an important role in the decision to seek healthcare [24]. Efforts aimed at improving healthcare quality, and promoting compliance with the standard, ethical practices through providers’ continual education and training may foster provider-patient relationship, trust and individuals’ decision to seek care for an injury.

In South Africa, gang violence, crimes and alcohol use and abuse are rife, which contributes to neighbourhood insecurity [3,26,27], Some of these challenges date to the South African liberation struggle from racial segregation and exploitation, characterised by political and state-sponsored violence. Even though political violence has subsided, interpersonal violence remains and is further exacerbated by urbanisation and socio-economic disparities [2], As shown in this study, these factors not only contributed to injury perpetration but also hinders the injured from seeking or reaching injury care. Due to fear of being attacked, injured persons were reluctant to travel to the nearest health facility for care and were delayed because ambulance drivers relied on police availability and escort for security purposes. The high crime and injury rates also lead to overcrowding of healthcare facilities, long waiting times and as shown in this study, discourage the injured from attempting to seek care. A similar impact has been reported on HIV care utilisation among women in SA [28].

To mitigate barriers related to gang violence, crimes and alcohol use and the consequential impact on injury care, a multisectoral approach is required. Community involvement, awareness creation and training are important first steps towards crime and violence reduction and timely injury care. For instance, studies in SA and other countries have demonstrated the positive impact of community coalitions and training of community emergency first aid responders in crowd control during violent injuries, provision of first aid care and ensuring timely transportation of the injured to health care facilities [29,30,31]. Other approaches including violence prevention peer-mentorship programmes [32] and alcohol regulation policies [15,33] shown to reduce violence, alcohol abuse and injury may also improve seeking injury care.

Importantly, our study showed that participants who experienced issues in one SDoH domain were likely to have experienced barriers in at least one other. For example, barriers related to healthcare access and quality strongly overlapped with neighbourhood and environment, and the social and community context SDoH domain barriers. This shows that most injured people are likely to be affected in care-seeking by multiple and potentially reinforcing social determinants. That we have found that SDoHs are so intertwined with accessing care after injury contributes to inequities in accessing care. That the social determinants are seen in many of the domains – not just economic or healthcare access and quality suggests that solutions to improve access to quality care after injury extend beyond providing Universal Health Coverage and that a multisectoral approach is needed to address social determinants of access to care for the injured.

## Strengths and Limitations

To the best of our knowledge, our study is the first to look at social determinants of health as barriers to seeking and reaching injury care in South Africa. The use of several data collection methods including IDIs and FGDs yielded more robust data and adds credence to our findings. Although our study was limited to the Western Cape Province of South Africa, alcohol abuse, gangs and violence are common social issues in other South African provinces [34,35,36]. Lastly, previously injured individuals who received care were recruited, thus our findings may have been limited by the inability to explore views from those who did not present to the formal healthcare facilities.

## Conclusion

While barriers to injury care best studied have related to factors around the provision of care, our study demonstrates that many factors outside of the health facility also impact access to injury care. We found a substantial role of neighbourhood, social, community, and cultural factors in seeking and reaching injury care and that these factors are multiple and reinforcing. Therefore, efforts aimed at improving access to injury care and outcomes should go beyond addressing health facility factors, must target creating a safe neighbourhood and should involve collaborations with multiple sectors including the community, the police, the transport department and alcohol regulation agencies.
